# Modeling Battery Behavior on Sensory Operations for Context-Aware Smartphone Sensing

**DOI:** 10.3390/s150612323

**Published:** 2015-05-26

**Authors:** Ozgur Yurur, Chi Harold Liu, Wilfrido Moreno

**Affiliations:** 1 Department of Electrical Engineering, University of South Florida, Tampa, FL 33620, USA; E-Mails: oyurur@mail.usf.edu (O.Y.); wmoreno@usf.edu (W.M.); 2 School of Software, Beijing Institute of Technology, Beijing 100081, China

**Keywords:** smartphone battery modeling, power efficiency, sensory operation modeling

## Abstract

Energy consumption is a major concern in context-aware smartphone sensing. This paper first studies mobile device-based battery modeling, which adopts the kinetic battery model (KiBaM), under the scope of battery non-linearities with respect to variant loads. Second, this paper models the energy consumption behavior of accelerometers analytically and then provides extensive simulation results and a smartphone application to examine the proposed sensor model. Third, a Markov reward process is integrated to create energy consumption profiles, linking with sensory operations and their effects on battery non-linearity. Energy consumption profiles consist of different pairs of duty cycles and sampling frequencies during sensory operations. Furthermore, the total energy cost by each profile is represented by an accumulated reward in this process. Finally, three different methods are proposed on the evolution of the reward process, to present the linkage between different usage patterns on the accelerometer sensor through a smartphone application and the battery behavior. By doing this, this paper aims at achieving a fine efficiency in power consumption caused by sensory operations, while maintaining the accuracy of smartphone applications based on sensor usages. More importantly, this study intends that modeling the battery non-linearities together with investigating the effects of different usage patterns in sensory operations in terms of the power consumption and the battery discharge may lead to discovering optimal energy reduction strategies to extend the battery lifetime and help a continual improvement in context-aware mobile services.

## 1. Introduction

New generation mobile devices with sensing capabilities, such as smartphones and tablets, will constitute a significant part of future mobile technologies. By reconfiguring and repurposing built-in sensors, these mobile devices can provide highly proactive services within the concept of human-centric or participatory sensing, which support computationally pervasive and emerging context-aware mobile applications. However, a major challenge standing up to these sensor-rich devices is resource limitation in terms of power, memory and bandwidth as compared to the capabilities of PCs and servers. In this sense, the design of mobile device-based context-aware middleware needs not only to create abstract models for the representation of the interested phenomena for the application services, but also to exploit the heterogeneous and unobtrusive physical world, as well as providing energy-efficient optimal sensor sensing and actuating solutions.

Due to the ever-increasing computing power and hardware development in mobile devices compared to the slow growth observed in stored capacity of energy densities in the mobile device-based battery technologies, the use of mobile devices is constrained by battery lifetime. Especially, continuously capturing user context through sensors in a context-aware application imposes heavy workloads on computations and hardware, e.g., the processor and relevant hardware peripherals, which, in return, makes the battery drain rapidly. Thereby, topics, such as the extension of battery lifetime, estimation of energy delivery or battery discharge and optimal energy management, have drawn much research interest in mobile computing.

In this sense, the examination of non-linear battery behaviors becomes crucial in terms of creating optimal sensor management systems. The battery lifetime mostly depends on energy consumption rate, discharge profile, *i.e.*, usage pattern and battery non-linearities. At the high energy consumption rate, the effective residual battery capacity degrades and results in having a shorter battery lifetime. However, any precautionary change in the usage pattern could extend the battery lifetime. For instance, decreasing the average energy consumption rate at any point in time could be one of the changes. More importantly, the physical non-linearities in the batteries could recover the lost capacity in cases where less energy is consumed after an aggressive battery discharge. Thereby, topics of battery non-linearities, such as the rate capacity effect and the recovery effect, will be explained in detail.

To this end, this paper examines battery modeling to investigate its behaviors and non-linearities. Then, it continues by modeling the energy consumption of smartphone sensors, and it takes the accelerometer sensor as an example. Both simulation results and smartphone application models are provided to verify the effect of variant sensory operations on the battery discharge and non-linearities. In this way, the linkage between a change in the energy consumption profile of sensor operation and the battery behavior to respond this change is exposed. Then, a reward process is integrated to create a sensor management system. The system aims at benefiting from battery behavior with respect to variant sensor loads forced by smartphone applications to prolong the battery lifetime. Finally, three different sampling methods are proposed on the evolution of the given reward process to achieve energy efficiency while maintaining accurate application services. A smartphone application is implemented to validate the given sensor management system.

## 2. Related Work

This paper first studies battery modeling under the scope of the battery non-linearities with respect to variant battery discharge profiles. The battery behavior is not consistent with respect to energy required by the device, due to the fact that the energy drawn from the battery is not always equivalent to the energy consumed by the device itself. In this regard, many different approaches have been used to model the battery properties, such as the electro-chemical models [1–3], electrical-circuit models [4] and analytical models [5,6]. However, these models provide the same battery lifetime for all load profiles, and they give better results for constant continuous loads, but not for variant or intermittent loads. In this sense, stochastic models [7] are presented to model batteries in an abstract manner by discretization of the battery charge and creation of probabilistic transitions among discharge levels caused by variations in workloads. Other detailed studies on battery modeling can be found in [8–10].

In addition, many studies have been put forward to extend battery lifetime in mobile sensing. Accordingly, most works done so far emphasize setting a minimum number of sensors by a mobile application with fixed duty cycles, using different deterministic sampling period schemes and maximizing power efficiency by solely applying less complexity in computations or by changing the transferring methods of data packets [11,12]. However, the impact of different usage patterns on devices needs to be investigated to link the projected effect on the power consumption. Especially, modeling the battery non-linearities together with an understanding of the relation between energy-wise usage patterns and battery depletion may lead to discovering optimal energy reduction strategies in order to help a continual improvement in context-aware mobile services.

Second, this study suggests opportunistic power saving methods at the low-level sensory operations in mobile sensing, so that battery lifetime could be extended. To be able to accomplish that, the energy consumption behavior of smartphone sensors is analytically modeled in this paper. Significantly, a smartphone application is carried out for the accelerometer sensor to investigate this behavior in detail. According to the proposed model, energy consumption profiles are created by assigning different pairs of duty cycles and sampling frequencies in sensory operations. Third, a Markov reward process is integrated to evaluate the energy consumption profiles and to represent the energy cost caused by each profile as an accumulated reward. The accumulated reward is also linked to the battery modeling to make a connection between the energy-wise usage pattern on sensors and battery behavior. Finally, with an understanding of non-linearity observed on the batteries in response to variant sensory operations, a fine efficiency in power consumption is achieved by proposing three different methods on the evolution of the reward process, while employing a human activity recognition (HAR)-based context-aware smartphone application.

The outline of the paper is as follows: Section 3 provides the battery modeling, which is very feasible to project onto mobile device batteries. Section 4 models sensory operations in terms of energy consumption and creates different load profiles during the operations to find out their effects on the total energy cost by taking the smartphone accelerometer sensor as an example. Section 5 makes a connection between battery non-linearity and sensor utilization to analyze battery discharge profiles triggered by energy cost occurring during sensory operations. Finally, Section 6 uses this connection to examine changing sensory operations to achieve a fine achievement in energy efficiency by providing a HAR-based context-aware smartphone application.

## 3. Smartphone Battery Modeling

A battery consists of electro-chemical cells, which drive electro-chemical reactions in order to convert chemically-stored energy into electrical energy. A cell includes two electrodes, which are an anode and a cathode, and the electrolyte, which separates these electrodes. The electrolyte may be liquid, as in lead-acid batteries, or solid, as in lithium-ion batteries. Lithium-ion batteries are widely used in notebook computers and cellular phones due to their high energy density and light weight.

An ideal battery has a constant voltage throughout a discharge regardless of the rate of the load, where the voltage is expected to drop instantaneously to zero when the battery is fully discharged. Therefore, the theoretical capacity of a battery is in the measure of its maximal charge. However, the specific energy that is delivered by a battery in practice is lower than the theoretically-defined energy of its active materials [13]. This is because the average voltage during the discharge is lower than the theoretical bound; also, the battery is not discharged to zero volts, and all available capacity is not utilized. The ions at the anode diffuse into the cathode when a current is drawn from the battery. If the current drawn is too high, the speed of diffusion is slower than the rate of ions reacted at the cathode. This results in a reduction that occurs at the outer surface of the cathode, and access to the inner ions is impossible. Hence, a drop in the output voltage sourced by the battery is observed. As the intensity of the current drawn increases, the loss of capacity due to the non-uniform deviation in ion concentration becomes significant, and therefore, the cell voltage decreases, which is called the rate capacity effect [14]. At a lower current drawn or when the discharge process occurs in some intermediate or periodic time scale, the ions could have enough time to diffuse into the inner cathode and let the charge recovery process take place. Therefore, the reacted ions at the cathode become uniformly distributed. This non-linearity is called the recovery effect, which depends on the discharge profile and the time length while no load is applied [15].

Let *C* and *Ć* be considered as the theoretical and nominal capacity values of a fully-loaded battery. In addition, let *x*(*t*) denote the level of available charge and *v*(*t*) denote the level of available theoretical capacity of the battery with the conditions of *t* ∈ [0, *T*] and (*v*(0),*x*(0)) = (*C*, *Ć*).

**Figure 1:**
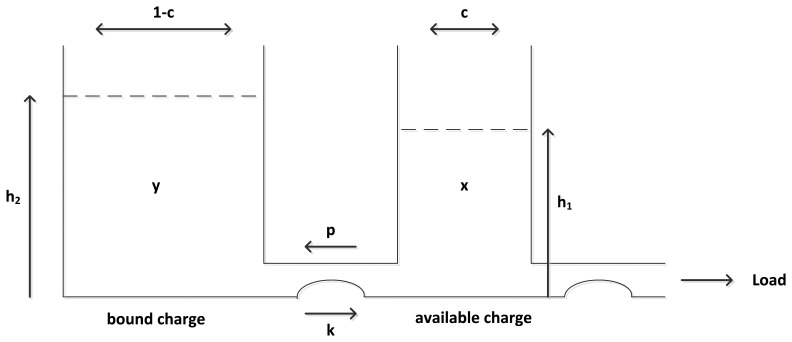
The two-well kinetic battery model (KiBaM).

The kinetic battery model (KiBaM) is the most powerful analytical battery model represented firstly in [16] and widely used in recent studies of [9,17,18]. KiBaM considers the stored charge to be distributed over two wells: the available charge well and the bound charge well, as shown in Figure 1. The bound charge well supplies electrons only to the available charge well; whereas, the available charge well supplies electrons directly to the connected load. The rate of charge flow between two wells depends on the conductance parameter *k* and the difference in heights of two wells, *h*_1_ and *h*_2_. The capacity ratio is denoted as parameter c, and it corresponds to the friction of total charge stored in the available charge well. Recall that *x*(*t*) denotes the available capacity of the battery and *v*(*t*) denotes the total capacity, where *x*(0) = *Ć* and *v*(0) = *C* > *Ć*, so *y*(*t*) = *v*(t) − *x*(*t*) becomes the bound charge.

On the other hand, KiBaM was primarily developed to model lead-acid batteries. Since these type of batteries have a flat discharge profile, there are some shortcomings of KiBaM to model as lithium-ion batteries, which are widely used in today's mobile devices. However, KiBaM could be still examined under some issues, such as battery lifetime, capacity rate and recovery effect [9,18]. To be able to extend the battery model for lithium-ion cells, a solid state diffusion must be added into KiBaM. In the solid state, electro-activated species at the electrodes experience drift motion diffusion in addition to random diffusion [19]. The drift motion can be added into flux as a negative charge, *p*, *i.e.*, degradation on the conductance parameter *k*.

During the battery operation, the current drawn due to discharge is denoted by *i*(*t*) ≥ 0 with the average discharge rate of $\hat{\lambda }=\lim_{t\to \infty }\frac{1}{t}\int_{0}^{t}i\left(s\right)ds$, if the limit exists. Then, the unit change in the charge stored in both wells is given by the following differential equations:

(1) $$\begin{array}{ll} \frac{dx\left(t\right)}{dt} & =-i\left(t\right)+k\left(1-p\right)\left(h_{2}-h_{1}\right)\\ \frac{dy\left(t\right)}{dt} & =-k\left(1-p\right)\left(h_{2}-h_{1}\right) \end{array}$$

with initial conditions of *x*(0) = *c*.*C* = *Ć*, *y*(0) = (1−*c*).*C* = *C*−*Ć*, *h*_1_ = *x*(*t*)/*c* and *h*_2_ = *y*(*t*)/(1−*c*).

When the battery supplies a continuous discharge, the available charge well would reduce rapidly; the difference of heights in both wells would grow, since there is no time to move the charge from the bounded charge well into the available charge well, and the battery would not last long. However, when the load is removed, a remedy charge flows from the bounded charge well into the available charge well until *h*_1_ and h_2_ are balanced. This gives the idea of why the recovery process is taking place when an intermittent charge is applied by variant loads.

By using Equation (1), the total discharge process becomes independent of the charge flow gradient:

(2) $$\begin{array}{cc} v\left(t\right)=x\left(t\right)+y\left(t\right)=C-\int_{0}^{t}i\left(s\right)ds, & t\geq 0 \end{array}$$

If a cell is subject to variant loads, then the current drawn would vary with different discharge rates by examining Equation (2) accordingly:

- Case 1: The constant discharge, Λ(*t*) = λ*t*, where *v*_λ_(*t*) = *C* − λ*t* on the bivariate dynamic system of (*v*_λ_(*t*), *x*_λ_(*t*)):
  (3) $$\begin{array}{ll} x_{\lambda }\left(t\right) & =cv_{\lambda }\left(t\right)-C_{\lambda }\left(1-e^{-k\left(1-p\right)t/c\left(1-c\right)}\right)\\ C_{\lambda } & =\lambda c{\left(1-c\right)}^{2}/k\left(1-p\right) \end{array}$$
- Case 2: The periodic regularly-spaced pulsed discharge, $\wedge \left(t\right)=\lambda r\sum_{j=1}^{t/r}\delta_{jr}$, where δ and λ*r* denote pulses and the released charge during the two consecutive pulses in *r* > 0 time intervals on the system of (*v*^(^*^r^*^)^(*t*), *x*^(^*^r^*^)^(*t*)):
  (4) $$\begin{array}{ll} x^{\left(r\right)}\left(t\right) & =cv^{\left(r\right)}\left(t\right)-\left(1-c\right)\lambda r\overset{t/r}{\underset{j=1}{\text{∑}}}e^{-k\left(1-p\right)\left(t-jr\right)/c\left(1-c\right)}\\ & =cv^{\left(r\right)}-C_{\lambda }^{\left(r\right)}\left(1-e^{-k\left(1-p\right)\left(C-v^{\left(r\right)}\right)/\lambda c\left(1-c\right)}\right) \end{array}$$
  with the evaluation of geometric sum; $C_{\lambda }^{\left(r\right)}=\lambda r\left(1-c\right)/\left(1-e^{-k\left(1-p\right)r/c\left(1-c\right)}\right)$ gives the bursting points of discharge profile due to periodically-drawn load.

**Figure 2:**
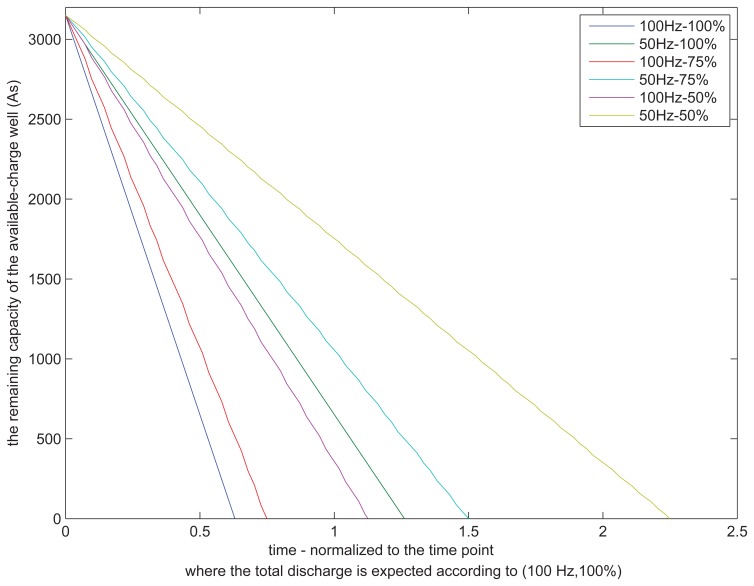
The KiBaM discharge model where *C* = 1400 mAh = 5040 As, *c* = 0.625, *k* = 4.5*E*^−5^/*s*, *p* = 0.1, λ = 2*f_s_*, *f_s_* = {50, 100} Hz, *r* = *n*Δ*t*, *n* = {1/2, 3/4,1}.

Figure 2 shows an example investigating KiBaM behavior under different load profiles and also at fixed system parameters. The load profiles are characterized by mixture pairs of sampling frequency, *f_s_*, and duty cycling on the load. Thereby, the load is defined by λ = 2*f_s_* where *f_s_* = {50, 100} Hz and *r* = *n*Δ*t* where *n* = {1/2, 3/4, 1}. For instance, if *n* = 1, this means the load has a constant discharge profile, *i.e.*, 100% duty cycling, whereas if *n* ∈ {1/2, 3/4}, this means duty cycling values of {50%, 75%} are applied on the load. In addition, the same power consumption rate per unit time is considered during discharge. On the other hand, the battery parameters for KiBaM are chosen as in *C* = 1400 mAh, c = 0.625, *p* = 0.1 and *k* = 4.5 *E*^−^*^5^*(1/*s*). These parameters can differ from one battery to another. However, with this example, it is intended to see how a battery discharges differently with respect to variant load profiles.

Figure 2 is normalized to the time point where the total discharge is expected according to the full depletion in *C* where *f_s_* = 100 Hz and 100% for the duty cycle. According to the results, obtaining a depletion time of less than one means that the battery seems as if it were depleted even though it still has charge in storage. In contrast, the battery lifetime is extended when the depletion time is greater than one. As a conclusion, two hypotheses can be made from the example. First, the constant aggressive loading by Equation (3) affects the discharge profile severely and yields to depletion of the device battery faster, even if the battery still has a sufficient stored charge. The second hypothesis by Equation (4) is that the battery recovery effect takes place when load gets lighter. The effect increases the available charge well and prolongs the battery lifetime.

## 4. Modeling Sensory Operation for Energy Consumption

By knowing how constant and intermittent loads affect battery depletion, sensor operations will be modeled in this section by introducing different sensor settings using variant sampling frequencies and duty cycles. The linkage between battery behavior and variant sensor operations by this model is supported and validated with simulations and experiments from a real smartphone application.

Assume that the operation of mobile device-based sensors is modeled by some certain time parameters, such as wake-up/initialization/termination time, *t_w_*, total time per sampling, *t_s_*, total time for operation cycle, *t_c_*, and total time throughout sensor run, *t_r_*. In addition, Ω*_s_* and Ω*_i_* are given as constant default sensor properties for power consumption to make a sampling and to run idle, respectively [20]. Mobile device sensors, such as accelerometer and the microphone, could be ruledby this generalized model, since they do not follow dedicated protocols to run, like GPS, Wi-Fi and Bluetooth do.

DC stands for the duty cycle that indicates an active sensory operation (*i.e.*, the time interval in which actual sensor samplings are being made) within *t_cycle_*. Thus, the number of sampling occurrences is found by *N_s_* = DC*t_c_*/*T_s_* where 0 ≤ DC ≤ 1, and *T_s_* is the sampling period (*i.e.*, inversely proportional to the sampling frequency, 1/*f_s_*) that defines a waiting time between two consecutive sensor samplings.

Then, a total energy consumption throughout a sensor's run is approximated by Equation (5), while ignoring power consumption wasted during the initialization and termination phases of the sensor.

(5) $$\begin{array}{ll} \Theta_{\text{total}} & \approx \left[\left(DC*t_{c}\right)\left(\overset{N}{\underset{k=1}{\text{∑}}}\left(\overset{\left(k-1\right)Ts+t_{s}}{\underset{\left(k-1\right)Ts}{\text{∑}}}\Omega_{s}+\overset{kTs}{\underset{\left(k-1\right)Ts+t_{s}}{\text{∑}}}\Omega_{i}\right)\right)+\left(\left(1-DC\right)*t_{c}\right)\Omega_{i}\right](\frac{t_{r}-2t_{w}}{t_{c}})\\ & \approx \left[N_{s}t_{s}\Omega_{s}+\left(t_{s}-N_{s}t_{s}\right)\Omega_{i}\right](\frac{t_{r}-2t_{w}}{t_{c}}) \end{array}$$

It would be hard to quantify and analyze the power efficiency actually achieved based on the provided battery model if we used sensors, such as Wi-Fi, 3G cellular connectivity or GPS, which apply certain design decisions, such as data transmission policies waiting for a specific medium to send and receive. Due to the reasons of suitability to easily match the derived cases for the battery discharge process, having a better realization and integration for energy consumption modeling that aims at using different sampling frequencies and duty cycles on sensory operations and general utilization in many smartphone applications, the accelerometer sensor is chosen for the implementation of a real-time application and simulations in the upcoming sections.

### 4.1. A Simulation Model

**Figure 3:**
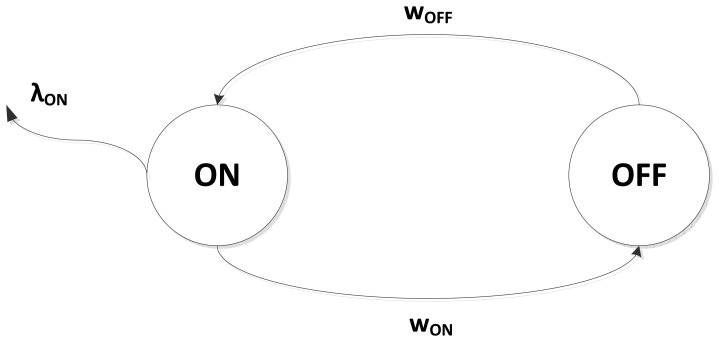
Interrupt Poisson process (IPP).

A sensory operation can be modeled as a one-burst process, also called ON-OFF sensory operation. This process is a special case of Markov-modulated Poisson processes. This special case is called the interrupted Poisson process (IPP) and has two states, ON and OFF, as shown in Figure 3. According to the process, the traffic is Poisson with a probability distribution of {*F*(*t*) = 1 − *e*^−λ^*^t^*}, but the traffic is generated at deterministic intervals in the ON state with a constant rate λ*_on_* and a length of mean time, *i.e.*, transition time, 1/*w_on_*. In contrast, there is no traffic in the OFF state, and the intensity of residency in this state is given by *w_off_*. The traffic in the IPP can be seen as a discrete event simulator for samplings in sensory operations. Thus, the intensity rates become λ*_on_* = *fs*, *w_on_* = (DC * *t_c_*)^−1^ and *w_off_* = ((1 - DC) * *t_c_*)^−1^.

Note that experiments in the upcoming section are carried out by implementing a context-aware application on a Blackberry RIM Storm II 9550 smartphone. Storm II consists of a three-axis accelerometer named ADXL346 from Analog Devices (see Table 1 and [21] for technical details). From the table, only drain current value *I_DD_* is an effective modeling parameter to determine power consumption through the sampling channel under a stable drain voltage *V_DD_*, since the power is formulated as *P* = *V* × *I* in physics.

By using the IPP sensor modeling together with the data obtained through Table 1 and Equation (5) with a parameter of *t_c_* = 2 s, the power consumption ratio in the sensor drain per each operation cycle under variant DC and *f_s_* values is shown in Table 2. According to the results, the smartphone accelerometer sensor consumes 4.45-times more power per each operation cycle in the most aggressive sampling mode in comparison with the least aggressive sampling mode.

### 4.2. An Application Model

The accelerometer sensor needs to be modeled in order to examine the power efficiency achieved under different sampling and duty cycling strategies. By using Equations (1) and (5), the power consumption model within each *t_c_* can be considered as in:

(6) $$\Theta_{t_{c}}=\sum_{0}^{\text{DC}*t_{c}}\left(L_{s}\left(x,y\right)-R_{s}\left(x,y\right)\right)+\sum_{\text{DC}*tc}^{tc}\left(L_{i}\left(x,y\right)-R_{i}\left(x,y\right)\right)+\sum_{0}^{tc}\left(L_{b}\left(x,y\right)\right)$$

where:

- (*x*, *y*) are bivariate dynamic battery system parameters that define capacity values stored inside the bound charge well and the available charge well in KiBaM.
- *L_s_* and *L_i_* are denoted as the discharged load when sensor samplings are being made and the sensor is idle, respectively.
  – If DC = 1, then the sensor makes samplings continuously This means the effect of *L_i_* can be ignored due to the constant discharge profile. Recall that the model follows Equations (3).
  – If DC ∈ [0,1), then the sensor makes samplings in defined intervals. In this case, the effect of *L_i_* cannot be ignored, so that the model follows Equations (4).
- *R_s_* and *R_i_* are battery recovery effects in KiBaM to define the remedy charge flowing from the bound charge well to the available charge well when sensor samplings are being made and the sensor is idle respectively.
- *L_b_* stands for the background load occurring instantly within smartphone operation. For instance, while experiment applications are running, the device is left in stand-by mode and only connected to a 3G network. There is no other system functionality being executed by the user while the sensor operates.

The application runs until the initially fully-charged one-year-old 1400-mAh smartphone battery depletes. Only one constant pair of sampling frequency and duty cycle is applied as operation parameters to the accelerometer sensor at each application run. Sampling intervals are modeled as *I_i_* = *n*/*f_s_* where *n* and *f_s_* are an integer value and sampling frequency, respectively. *I_i_* defines a waiting time between two consecutive sensor samplings; *n* = {1, 2, 4, 8} and *f_s_* = 100 Hz are taken. On the other hand, values of {0.5, 0.75, 1} are taken for DC. *t_c_* is also taken as 1 s.

**Figure 4:**
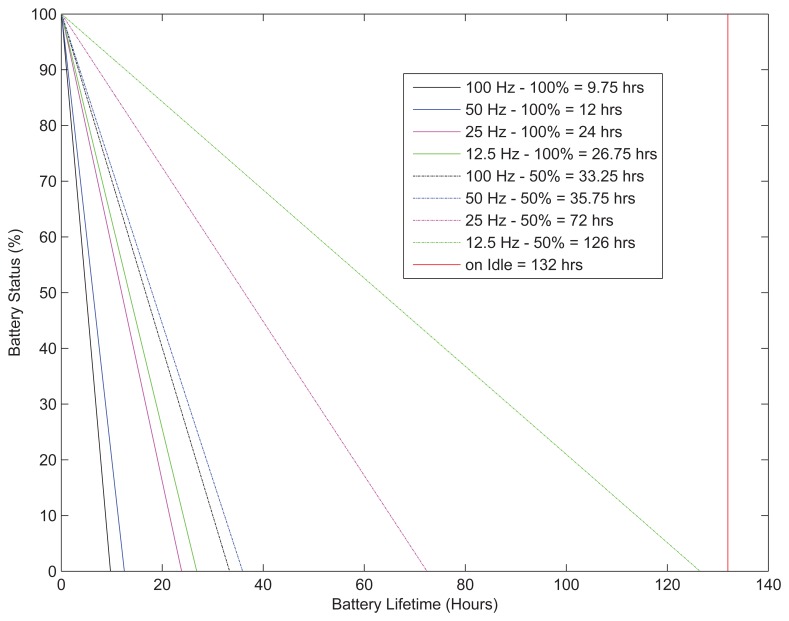
The battery depletion due to variant sampling frequencies and duty cycles within the operation of the accelerometer sensor (samples are taken at every 20 min).

Results are shown in Figure 4. Note that the Blackberry Java 7.1. SDK only helps to reveal the remaining battery status. According to the results, applying a more aggressive sampling methodology causes the battery to deplete more quickly. In addition, the lower value of DC makes the battery recover effect more significant and, thus, prolongs the battery lifetime.

According to Equation (5), energy consumption per time of 1/*fs*_max_, (= 1/100), under different sampling frequency is modeled as in:

(7) $$\Theta_{n*12.5Hz}=n*\Omega_{\text{sample}}+\left(n_{\text{max}}-n\right)*\Omega_{\text{idle}}$$

Then, Equation (7) together with Equation (6) turn into:

(8) $$\Theta_{t_{c}}=\frac{\text{DC}*t_{c}}{n_{\max}/fs_{\max}}\left(\Theta_{n*12.5Hz-r_{s}}\right)+\frac{\left(1-\text{DC}\right)*t_{c}}{1/fs_{\max}}\left(\Omega_{\text{idle}}-r_{i}\right)+l_{b}$$

where *r_s_*, *r_i_* and *l_n_* are considered to have a stable energy consumption rate per unit time.

### 4.3. Comparison: Simulation vs. Application

Since parameters *r_s_* and *r_i_* cannot be verified through smartphone programming, to compare energy consumption between the analytical model and the application, the following inequality needs to be approximately satisfied:

$$\frac{\Theta_{100Hz,\text{DC}=1}^{\text{real}}}{\Theta_{100Hz,\text{DC}=1}^{\text{sim}}}\approx \frac{\Theta_{50Hz,\text{DC}=1}^{\text{real}}}{\Theta_{50Hz,\text{DC}=1}^{\text{sim}}}\approx \frac{\Theta_{25Hz,\text{DC}=1}^{\text{real}}}{\Theta_{25Hz,\text{DC}=1}^{\text{sim}}}\approx \frac{\Theta_{12.5Hz,\text{DC}=1}^{\text{real}}}{\Theta_{12.5Hz,\text{DC}=1}^{\text{sim}}}$$

The effect of *r_s_* and *r_i_* becomes more significant when DC ≠ 1. Hence,

$$\frac{\frac{1}{9.75}-\frac{1}{132}}{\Theta_{100Hz,\text{DC}=1}^{\text{sim}}}\approx \frac{\frac{1}{12}-\frac{1}{132}}{\Theta_{50Hz,\text{DC}=1}^{\text{sim}}}\approx \frac{\frac{1}{24}-\frac{1}{132}}{\Theta_{25Hz,\text{DC}=1}^{\text{sim}}}\approx \frac{\frac{1}{26.75}-\frac{1}{132}}{\Theta_{12.5Hz,\text{DC}=1}^{\text{sim}}}$$

The inequalities with a small error margin prove a valid connection between the analytical model and the application in terms of the power efficiency achieved. Relevant error would stem from computational workload on the processor and non-linear functionality of the battery. With the help of these facts, the accelerometer sensor is analytically modeled with sufficient success. This information reveals that many sensors whose operation method is similar to the accelerometer, such as the microphone, gyroscope, pulse meter and so forth, can be modeled in a similar way.

## 5. Modeling Smartphone Sensory Operations

This section presumes ongoing sensory operations in a smartphone application, and their effects on the battery depletion can be modeled as a semi-Markov reward process. The changes in sensory operation in terms of sampling frequency and duty cycling makes it semi-Markovian, since the time spent in any sensor setting and its transition to another sensor setting is random, which will be based on the accuracy ratio defined in the next section. Reward is associated with the proposed KiBaM battery model-based power consumption or affected battery depletion by an active sensor setting.

Assume that a set of DC and a set of fs are given by {1, 0.75, 0.5} and {100, 50, 25, 12.5} Hz, respectively. In addition, let us define a state space *S* that lies over these two datasets as in *S* = {*S*_DC_ × *S_fs_*} where ∀*j* ∀ DC, ∀*k* ∈ *fs*, {*j*, *k*} → {*i*} and *i* ∈ *S.* Thus, the state space represents sensor settings or battery discharge profiles caused by different sensor sampling operations with states of {*S*_{1,100}_ = 1, *S*_{1,50}_ = 2, …, *S*_{0.75,100}_, …, *S*_{0.5,12.5}_ = N}.

Let us also define a power consumption rate, ψ, for each state and assume that it may vary in time. Hence, a total accumulated power consumption, *v*, while residing in state *i* from time *s* to *t* is introduced by the difference equation of:

(9) $$\Delta_{t}\left(v_{i}\left(s,t\right)\right)=\psi_{i}\left(t,v_{i}\left(s,t\right)\right)$$

with the probability distribution of *F^V^*(*t*, *v*) = *Pr*{*V*(*t*) < *v*}.

According to KiBaM, ψ could be two different types. The first type represents an actual power consumption rate, ψ*_i_*_,1_, consumed by sensory operation settings defined by state *i*; whereas, the second type, ψ*_i_*_,2_, belongs to degradation in maximum battery capacity, *i.e.*, the recovery effect that makes a remedy charge flow migrate from the bound charge well into the available charge well. By evaluating Equations (1) and (9) together, KiBaM equations are correlated with the accumulated power consumptions by:

(10) $$\begin{array}{ll} \Delta_{t}\left(v_{i,1}\left(s,t\right)\right) & =\psi_{i,1}\left(v_{i,1}\left(s,t\right),v_{i,2}\left(s,t\right)\right)\\ & =I_{i}-k\left(1-p\right)\left(h_{2}-h_{1}\right)\\ \Delta_{t}\left(v_{i,2}\left(s,t\right)\right) & =\psi_{i,2}\left(v_{i,1}\left(s,t\right),v_{i,2}\left(s,t\right)\right)\\ & =k\left(1-p\right)\left(h_{2}-h_{1}\right) \end{array}$$

where *h*_2_ > *h*_1_ > 0 and *v_i_*_,1_(*s*, 0) = *v*_i,2_(*s*, 0) = 0.

The joint distribution of the accumulated battery discharge during state *i* becomes:

(11) $$F_{i}^{\left(V_{1},V_{2}\right)}\left(t,v_{i,1},v_{i,2}\right)=\mathrm{Pr}\left\{S\left(t\right)=i,V_{1}\left(t\right)\leq v_{i,1},V_{2}\left(t\right)\leq v_{i,2}\right\}$$

with boundaries of min{*v_i_*_,1_, *v_i_*_,2_} = {0, 0} and max{*v_i_*_,1_, *v_i_*_,2_} = {*c.C*+*v_i_*_,2_, (1−*c*).*C*}, since the battery always has a predefined capacity *C* that is distributed by a fraction factor *c* over two wells in KiBaM.

Therefore, by using Equations (10) and (11), the battery gets empty when *V*_1_(*t*) ≥ *Ć* + *V*_2_(*t*):

(12) $$\mathrm{Pr}\left\{V_{1}\left(t\right)\geq Ć{+\text{V}}_{2}\left(t\right)\right\}=\sum_{v_{i,1}=0}^{Ć+v_{x}}\sum_{v_{i,2}=0}^{v_{i},x}\sum_{i\in S}F_{i}^{\left(V_{1},V_{2}\right)}\left(t,v_{i,1},v_{i,2}\right)$$

where *v_x_* ≤ *C* − *Ć*.

If *v_i,x_* ≠ *C* − *Ć* when the battery gets empty, it gives a clue that a constant high load has been applied to the battery, and this makes the recovery effect not take place.

The first power consumption rate, ψ*_i_*_,1_, for each state can be obtained by using Equation (5), Tables 1 and 2. The power consumption at each *fs* is proportional to flowing drain current, *I_DD_*, under a stable drain voltage supply. Thus, ψ*_i_*_,1_ can be defined as in:

(13) $$\psi_{\left\{\text{DC}=j,fs=k\right\},1}=\Theta_{\left\{\text{DC}=j,fs=k\right\}.}$$

On the other hand, the second power consumption rate, ψ*_i_*_,2_, can be derived from Equations (1), (3) and (4) if a state *l* transits into another state *i* at time *s*:

(14) $$\psi_{i,2}\left(s,t\right)=\frac{c\left(1-c\right)}{k}\left(\psi_{l,1}-\psi_{i,1}\right)\left(1-e^{-k\left(t-s\right)/c\left(1-c\right)}\right)$$

where *t* ≥ *s* and ψ*_i_*_,1_ > ψ*_i_*_,1_, since the recovery effect takes place whenever a lower load is proceeded.

To this end, assume that a semi-Markov chain represents the evolution of changing sensory operation methods. The chain consists of a finite state space *S* = {1,…, N}, the state transition density matrix *q* ∈ *Q* and the state transition matrix *p* ∈ *P*. *q* represents jump or transition rates from user state *i* to user state *j* at time *t*. Whenever *i* = *j*, this means that the current user state remains unchanged, or, *i.e.*, a dummy transition occurs. Furthermore, *p_il_*(*s*,*t*) = *Pr*(*S*(*t*) = *l* | *S*(*s*) = *i*) where *i*, *l* ∈ *S*, and *t* ≥ *s* represents a user state transition probability matrix, which accepts the relation of lim*_t_*_↓_*_s_∂p_il_*(*s*, *t*)/*∂t* = *q_il_*(*s*). The chain can revisit a user state at different system times, and also, not every user state needs to be visited. Hence, there is no requirement that user state transition probabilities must be symmetric (*p_il_* ≠ *p_il_*), or a specific state might remain in the same in the succession of time (*p_il_* = 0).

In addition, a reward structure can be attached to this on-going chain, and it can be thought of as a random variable associated with the state occupancies and transitions. The reward can be seen as power consumption per unit time while a mobile device battery is discharging; thereby, it is denoted by the same ψ, and *S* is then redefined as the battery discharge profiles/states. As a result, the total reward, *i.e.*, total power consumption, depends on the total visiting time in a state *i*. Then, it can be said that the reward ψ*_i_* belonging to state *i* is proportional to the aggregation of Equations (13) and (14).

Finally, the general evolution of a semi-Markov reward process to describe power consumption caused by sensory operations is attached to Equation (12) and given by:

(15) $$V_{l}\left(s,t\right)=V_{i}\left(s,t-1\right)+\sum_{l\in N}p_{il}\left(s,t-1\right)\psi_{l}\left(s,t\right)$$

where the left-hand side, *V* , represents the expected present value of all received rewards from time *s* to *t* given that process enters into state *i* at time *s*. Whereas, the first element of the right-hand side represents the aggregation of rewards earned both at the previous time, the second element of the right-hand side is the reward obtained from either continuity in the same state or transition to another state.

## 6. Performance Evaluation

A human activity recognition (HAR)-based context-aware application is examined to investigate the power efficiency caused by the sensor utilization with respect to the recognition accuracy in the interested context. Our previous works [22,23] are adopted for context recognition by using the smartphone accelerometer, and accordingly, some user postures, such as sitting, standing, walking and running, are chosen as interested context. A Blackberry RIM Storm II 9550 smartphone with a fully-charged 1400-mAh battery is used for experiments. Sensor settings and the associated reward process were already explained in the previous section. In this section, a set of rules is applied by introducing an action space for this process, and methods are proposed for the transitions among sensor settings through the evolution of the underlying process.

For performance analysis, a similar user activity profile is performed by applying two proposed sensory sampling methods differently on a HAR-based context-aware application with a sufficient number of experiment repetitions (over 20 experiments for each sensor management method with variant users). Accordingly, a user profile begins with sitting and then standing each for 30 s (used for calibration); then, it transits into another posture randomly at the end of the following visiting times of {5, 10, 30, 60,100, 300} s. The adopted recognition algorithm uses the smartphone accelerometer and recognizes the defined postural activities with a period of one second. In the first one-minute runtime, the accelerometer sampling parameters are set to {DC = 100%, *f_s_* = 100 Hz}. Then, sensory sampling parameters are adaptively changed by our following proposed methods.

An action state space, *a* = {decrease = 1, preserve = 2, increase = 3} ∈ 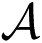, is defined to regulate sensory operations, *i.e.*, to decrease, preserve and increase power consumptions, respectively, while maintaining the application accuracy in context awareness. A 10% threshold value is defined for the accuracy margin to represent the tolerance where false recognitions are observed. Depending on the ratio of false recognitions, actions are applied. This ratio is calculated every 10 s. If the ratio of false recognitions occurring within the recent 10-s of application time is lower than the specified margin, then Action 2 is taken to preserve the same setup for the applied sensory operations. In addition, if the ratio hangs in the same margin at least for a sufficient time *t_suff_*, which is set to 20 s for the experiments, then Action 1 is taken to reduce power consumption by estimating that the observed postural activity is expected to stay on hold. In contrast, if the ratio of false recognitions is higher than the specified margin, then Action 3 is taken to increase the power consumption in sensory operations by making more aggressive samplings. Thereby, a fine balance between the power consumption caused by the sensory operations and the application accuracy is attempted.

There are three different state transition methods applied for the Markov reward process defined by Equation (15). The methods control power consumption by changing DC and/or *f_s_* according to the recognition accuracy observed in postural activities. Relevant adjustments are regulated by the action set of *a*.

### 6.1. Method 1

This method tries to change DC in the first place, rather than changing *f_s_*, and proposes how to wander over the defined space *S* according to actions *a* by:

(16) $$S_{\left\{j,k\right\}}\left(t\right)=\{\begin{array}{ll} S_{\left\{j-1,k\right\}}\left(s\right), & a=1,j\neq j_{\min}\\ S_{\left\{j,k-1\right\}}\left(s\right), & a=1,j=j_{\min},k\neq k_{\min}\\ S_{\left\{j+1,k\right\}}\left(s\right), & a=3,j\neq j_{\max}\\ S_{\left\{j,k+1\right\}}\left(s\right), & a=3,j=j_{\max},k\neq k_{\max}\\ S_{\left\{j,k\right\}}\left(s\right), & \text{otherwise} \end{array}$$

### 6.2. Method 2

This method, in contrast to Method 1, makes the adjustments in *f_s_* in the first place. Then, the relevant state transitions over *S* become:

(17) $$S_{\left\{j,k\right\}}\left(t\right)=\{\begin{array}{ll} S_{\left\{j,k-1\right\}}\left(s\right), & a=1,k\neq k_{\min}\\ S_{\left\{j-1,k\right\}}\left(s\right), & a=1,k=k_{\min},j\neq j_{\min}\\ S_{\left\{j,k+1\right\}}\left(s\right), & a=3,k\neq k_{\max}\\ S_{\left\{j+1,k\right\}}\left(s\right), & a=3,k=k_{\max},j\neq j_{\max}\\ S_{\left\{j,k\right\}}\left(s\right), & \text{otherwise} \end{array}$$

### 6.3. Method 3

State transitions are executed according to the ascending order of power consumption rates shown in Table 2. Hence, both DC and *f_s_* could be changed simultaneously by this method as in:

(18) $$S_{\left\{i\right\}}\left(t\right)=\{\begin{array}{ll} S_{\left\{i-1\right\}}\left(s\right), & a=1,i\neq i_{\min}\\ S_{\left\{i+1\right\}}\left(s\right), & a=3,i\neq i_{\max}\\ S_{\left\{i\right\}}\left(s\right), & \text{otherwise} \end{array}$$

**Figure 5:**
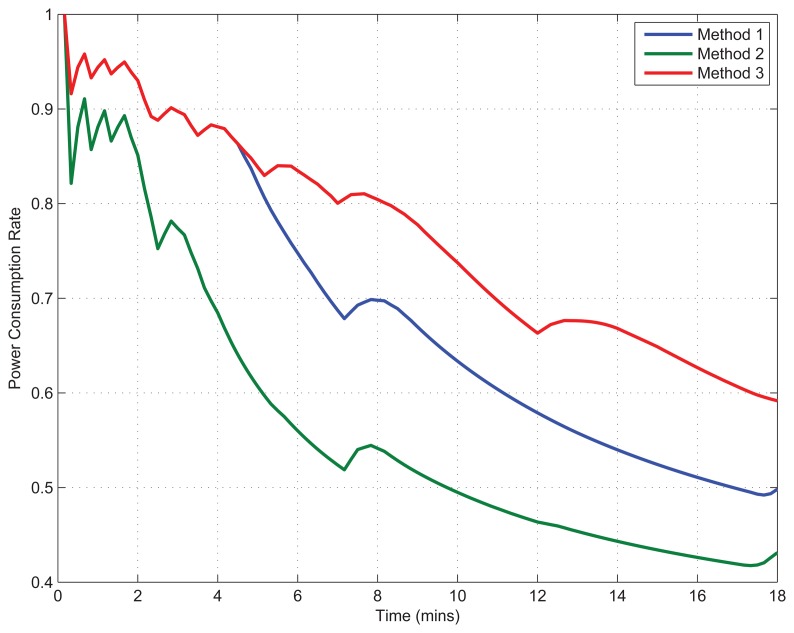
Power consumption ratio analysis in comparison to the aggressive sampling (DC = 100%, *f_s_* = 100 Hz) (results are averaged with respect to the experiments).

Each method given by Equations (16)–(18) is applied differently to the semi-Markov reward process for the similar user activity profile within the same experimental setups in order to analyze the power efficiency or battery life extension achieved by the smartphone accelerometer, shown in Figure 5, while keeping overall 10% accuracy loss.

During the first one-minute period, the aggressive sampling method is applied, which results in the highest recognition accuracy. After, state transitions are regulated by our proposed methods together with actions according to the ongoing user activity profile; therefore, we observe an accuracy decrease. The ups and downs depicted in Figure 5 show the increase of power consumption to compensate the worsening of application accuracy and the decrease of power consumption to make an opportunistic sensory operation adjustment, due to the stability observed in the application accuracy. On the other hand, a drastic decrease can be seen in the accuracy ratio initially, due to the changing sensor settings by adjusting a lower number of sensory samplings to infer the user activities; the accuracy ratio then increases, since a better adaptation is achieved by the user activity recognition algorithm when time progresses. Among the three proposed methods, a comparison can be found as “Method 3 > Method 1 > Method 2” in terms of the power consumption ratio. Accordingly, the sampling at slower frequencies consumes higher power than the sampling at lower duty cycles. However, this shows the opposite trend in terms of the recognition accuracy. This is because sampling at slower frequencies still obtains information about user activity, where the sampling at lower duty cycles cannot. On the other hand, Method 3 has the highest efficiency in the power consumption, since it switches sensory operation modestly, while achieving a fine recognition accuracy. In general, the tradeoff solutions achieve overall 50% enhancement in power consumption caused by the physical sensor work on the battery discharge, with respect to the overall 10% decrease in the accuracy ratio for user state recognitions. In addition, Figure 5 is scaled to the amount of battery discharge based on elapsed time, where the same experiment is carried out for the most aggressive sampling strategy.

## 7. Discussions

Since the use of smart devices is constrained by their limited battery lifetime and the slow growth in energy density provided by battery technologies, this drives the need for accurately modeling power consumption profiles. As studied in this paper, modeling the battery through KiBaM and associating it with existing hardware to achieve efficiency in power consumption can be classified under power optimization models based on mathematical estimation and the hardware operation/specific domain for mobile sensing. The diversity in architectural designs within smart devices and their components along with the differences in usage patterns present a challenge to profile the actual energy consumption. Therefore, many other existing research efforts are already been made to model power consumption profiles for smart phone use. Traditional methods [24] use external equipments, such as power meters, to model energy estimation based on measurements of device operation in different activity modes. In contrast, some studies, classified under system executing modeling [25,26], remove the necessity of using extra equipment by runtime monitoring to track key underlying operating system parameters and hardware components. They first determine a measurable power cost of using a specific device component and characterize its effect on battery drain over a unit of time. Then, they collect power consumption-related data through use of applications and break this data down into pre-measured component base statistics collected in applications in order to create a proper energy model. The final step aggregates all of the data collected by use of different applications and components and generates power cost coefficients to anticipate online power consumption or battery drain for future smartphone operations. Furthermore, this approach allows one to emulate the power consumption of mobile software architectures for optimization and leads to creating model-driven testing and auto-generated code, as well as specifying domain-specific modeling, by selecting a set of hardware components that satisfy both functional and nonfunctional requirements, while minimizing overall power consumption. However, in this paper, we intend to model the battery and application domain-specific sensor use to examine variant energy loads acquired by sensor operation and their effects on battery depletion and possible energy savings.

## 8. Conclusions

This paper investigates mobile device-based battery behavior with respect to variant sensory operations in a smartphone application. In this manner, the paper studies the battery non-linearities by examining an effective battery model, called KiBaM, for various battery discharge profiles. The paper also models sensory operations by providing the smartphone accelerometer as an example to analyze the linkage between battery discharge and power consumption caused by the sensor. With the understanding of non-linearity observed on the batteries with respect to variant operation methods in sensors, a fine efficiency in power consumption is achieved. Thereby, a Markov reward process is integrated to create energy consumption profiles in sensory operations and to represent the total energy cost by each profile as an accumulated reward. Finally, three different sensor employment methods are proposed on the evolution of the reward process while employing a human activity recognition (HAR)-based context-aware smartphone application. It is shown that with the integration of the battery non-linearities into the diverse operation methods in sensors, a fine power consumption balance is achieved while employing context-aware services in resource-constrained mobile devices.

## Figures and Tables

**Table 1:** The drained current vs. data rate in the accelerometer, ADXL346.

**(*V****_DD_* **= 1.8 V, *V****_S_* **= 2.6 V)**	

**Data Rate (Hz)**	*I_dd_*(μ*A*)
100	140
50	90
25	55
12.5	40

Autosleep Mode	23

Standby Mode	0.2

**Table 2:** The power consumption ratio in the sensor drain per each operation cycle: *t_c_* = 2 s; and the comparison applied based on (50%, 12.5 Hz).

**(DC**(%), *f_s_* **(Hz))**	**Ratio**
(100, 100)	4.45
(50, 100)	2.58
(100, 50)	2.85
(50, 50)	1.80
(100, 25)	1.75
(50, 25)	1.24
(100, 12.5)	1.26
(50, 12.5)	1
